# The Interim Report of the Fabian Society

**Published:** 1914-04-04

**Authors:** 


### NATIONAL INSURANCE ACT DEVELOPMENTS.

### The Interim Report of the Fabian Society (*continued*).

### THE SHORTAGE OF HOSPITAL ACCOMMODATION.

In connection with their remarks on the lack of provision for the serious illness of insured persons, the committee, which was instituted by the Fabian Research Department to investigate the working of the National Insurance Act, lay special emphasis in their report on the shortage of hospital accommodation—a fact to which we have repeatedly directed attention. We are told that the Commissioners hold that it is not incumbent on them or on the Insurance Committees to provide hospital treatment, and that it is assumed that the general practitioner will always be able to tell the patient whose case requires institutional treatment of some hospital to which he can gain admission. The committee regret to say that their investigation fails altogether to confirm this optimism. Case after case is reported in which, where admission has ultimately been obtained, the patient needing an operation has (if not a case of urgency) had to wait eight, ten, or thirteen weeks for a vacant bed. They find that the London hospitals have long "waiting lists," and that it is reported throughout the country that pressure on the hospital accommodation has been greatly intensified since the Act came into operation. Several causes are mentioned to account for this—causes that we have repeatedly pointed out.

They comment also on the astonishing fact that neither at the Commissioners' office nor at the Local Government Board is there any complete survey of hospital accommodation throughout the kingdom. From investigation they find that not one county in England has voluntary hospital beds equal to the low standard of 2 per 1,000 of the population, while many counties fall much below even 1 bed per 1,000 population. This lack of provision is contrasted with the provision made in Germany of from four to six times as many beds.

### The Cause of Protracted Sickness Benefit.

The committee conclude that this inability to get hospital treatment is a very potent cause of unnecessarily protracted sickness benefit, and that until this is remedied little hope can be entertained of a diminution of such claims upon the funds of the approved societies. They recommend that when sufficient hospital accommodation cannot be obtained for all insured persons, the county or county borough council should be empowered and required to provide additional hospitals, the cost being met to the extent, say, of 75 per cent., by a new grant in aid from Parliament.

Opinion may differ as to the precise form in which the State aid should be granted, but we are glad that so influential and impartial an inquiry has recognised the vital necessity, not only in the interests of those who are actually suffering, but also as regards the whole financial soundness of the Insurance scheme, of the voluntary hospitals, and that something must be done at once to give effect to the committee's recommendations.

### Lack of Expert Diagnosis.

Another feature of the administration of medical benefit which calls forth a serious condemnation from the committee is the lack of expert diagnosis. They state that serious complaints have been made by the approved societies that sufficient care is not given by the doctors to ascertain the real causes of the illnesses of insured patients, and that this is evidenced by the loose manner in which many of the certificates are filled in which give the insured persons the right to claim sickness benefit from their societies. The blame for this state of affairs is not altogether placed on the doctors. They are to a large extent precluded from giving that care and attention to the cases that come before them by force of circumstances. The panel doctor in our great cities frequently has to see as many as a hundred patients in an evening—so remark the committee as the result of their investigations—and it is no wonder, therefore, that, with the knowledge that large numbers of patients are waiting, the doctor must be content with a very superficial examination of each individual.

It is not only, however, that the doctors in many cases are so overworked that they cannot give the time for a proper examination that calls for comment. The committee are forced to the conclusion that outside the large towns, where the practitioners can call in the aid of the voluntary hospitals to assist them in making an expert diagnosis of the more difficult cases, the means rarely exist to enable the doctors to make the scientific diagnosis that should be made and which was evidently intended to be made. They insist emphatically upon the condition laid down by the Chancellor of the Exchequer, when he agreed to the additional Government grant to the doctors—namely, that the service was to be improved and that "where necessary the practitioner should resort to those modern methods of exact diagnosis the importance of which is increasingly recognised in the profession." In spite of this requirement little attention is paid to the necessity for obtaining precise information as to the diseases from which the insured persons are suffering, and the inevitable result is that in innumerable cases the treatment for symptoms only leads to prolonged sickness, and a consequent drain on the sickness benefit funds.

### The Provision of Drugs and Appliances.

The committee then proceed to criticise the arrangements that have been made as to the provision of drugs and appliances. The patients, it is true, seldom have any difficulty in getting the drugs that have been prescribed, nor is there any general ground for complaint as to the quality of the drugs supplied, but the committee question the legality of the regulations made by the Insurance Commissioners whereby the local Insurance Committees have instituted "Drug Funds" which limit the amount that can be spent on drugs. Their chief criticism is, however, directed against the method adopted for the payment of the chemists. By the disastrous device of allowing a "floating" sixpence per insured person which should be available if required, and only if required, to meet the payment of the chemists' bills in full, and if not required for this purpose to be available for distribution among the doctors, the latter have been furnished with an inducement to keep down the cost of the medicines and drugs which they prescribe to the lowest possible figure. This inducement to prescribe inexpensive drugs is emphasised by the limitation of the list of drugs that may be ordered without special formalities, although there has apparently been no direct prohibition of the use of remedies merely because they are costly.

In similar manner the list of surgical appliances that may be claimed as a part of medical benefit is lamentably deficient, and, in fact, the right of the Commissioners to draw up a rigid list of appliances is called in question. The committee hold, for instance, that it should be possible, under the provision made for the supply of surgical appliances by the Act, for an insured person to obtain a truss where this is ordered by the doctor as being necessary before the patient can resume work. As trusses, elastic stockings, and other similar appliances are now rigorously excluded from the list it happens in many cases that insured persons cannot obtain them, or, at least, may have to wait for some weeks until they have saved enough money to buy them, during which time they have unnecessarily been allowed to remain on the sickness benefit funds of their societies.

The committee conclude this section of their report by referring to the comparatively large number of insured persons who have as yet failed to avail themselves of the provision of medical benefit—inadequate though it be—that has been made for them. "It is significant," they say, "that they have not been able to ascertain that any diminution whatever has yet been noticed in the numbers of those resorting to the Poor Law. For all its vast expenditure, the Insurance Act, which comes to the aid of the artisan and the factory operative, still leaves unprovided for a vast mass of those for whom provision was most needed. There is much to be thought of when the treaty with the doctors (and the annual grant of nearly two millions, made only for three years) comes up during the year 1915 for reconsideration!"

### Administration of Sanatorium Benefit.

Proceeding to the investigation of the administration of sanatorium benefit the committee remark that, although the Act was passed in December 1911 and sanatorium benefit was the first of the four benefits now in force to come into operation, it is the most backward and least adequately organised. It is true that the Act does not make pretence to provide treatment in a sanatorium for every insured person who may be suffering from tuberculosis, though popular imagination was stirred by the promise of the "first-class hotels." The treatment provided by the Act is "in a sanatorium or other institution *or otherwise*," and full allowance must be made for the difficulties in providing the necessary accommodation. But the committee remark that after two years' organising work we are only at the beginning of the provision contemplated by the Act. According to a careful estimate they have made, it is calculated that there should be 1 bed for every 1,000 population for the use of tuberculous persons, which would mean 36,000 beds for England, or four times the present provision.

The most serious criticism, however, is that sufficient precautions have not been taken to ensure the earliest possible detection and notification of the disease, and that even when a case has been notified there may be long and anxious delay before the treatment—whether domiciliary or in a sanatorium—is given. Moreover, incurable cases are not furnished with treatment and are allowed to remain in their own homes as a source of infection to those about them.

The lack of organisation is attributed by the committee to the present division of authority for the administration of the benefit, and they suggest that drastic new measures are necessary to make the administration effective.

